# Multiple lineages in freshwater mytiloid bivalves: polyphyly of *Sinomytilus* and establishment of a new genus from China (Bivalvia, Modiolidae)

**DOI:** 10.3897/zookeys.1295.205333

**Published:** 2026-10-20

**Authors:** Hui Zheng, Xing-Xiao Wang, Ji-Wang Xie, Chun-Hua Zhou, Yue-Ming He, Hong-Quan Xiang, Shan Ouyang, Fang-Fa Zeng, Xiao-Ping Wu

**Affiliations:** 1 School of Life Sciences, Nanchang University, Nanchang 330031, China School of Life Sciences, Nanchang University Nanchang China; 2 College of Life Sciences, Capital Normal University, Beijing 100048, China College of Life Sciences, Capital Normal University Beijing China; 3 College of Engineering and Technology, Tianjin Agricultural University, Tianjin 300384, China College of Engineering and Technology, Tianjin Agricultural University Tianjin China; 4 Department of Animal Science, Yuxi Agriculture Vocation-Technical College, Yuxi 653106, China Department of Animal Science, Yuxi Agriculture Vocation-Technical College Yuxi China; 5 Jiangxi Provincial Ecological Environment Monitoring Center, No. 1131 Hongdu North Avenue, Donghu District, Nanchang City, Jiangxi Province, 330039, China Jiangxi Provincial Ecological Environment Monitoring Center Nanchang City China

**Keywords:** Ancient lake, freshwater adaptation, molecular phylogeny, morphological convergence, Mytiloidea, new genus, *
Sinomytilus
*, Yunnan Plateau

## Abstract

The systematics of freshwater mytiloid bivalves has long been confounded by morphological convergence, particularly among East Asian taxa traditionally assigned to *Sinomytilus*. In this study, an integrative revision of Chinese *Sinomytilussensu lato* was conducted, combining shell morphology, anatomical observations, multi-locus phylogenetic analyses, mitogenome-based phylogeny, and molecular dating. Our results demonstrate that *Sinomytilus* as currently defined is polyphyletic. *Sinomytilus swinhoei* from the Yangtze and Yellow river basins clusters with *Limnoperna* species rather than with the type species *S. harmandi* from Southeast Asia, whereas the small-bodied species from Lake Fuxian forms an independent lineage. Divergence time estimates indicate that the freshwater modiolid clade originated in the middle Jurassic (~173 Ma), coinciding with pronounced sea-level fluctuations around the Aalenian–Bajocian. Accordingly, a new genus is erected, *Shenzhousia* Zheng, Wang, He & Xiang, **gen. nov**. (type species: *Sinomytilus swinhoei*), and a new subfossil species described, *Shanomytilus frendae* Wang, Zheng, He & Xiang, **sp. nov**., from the Quaternary Luliang Basin. This study clarifies the taxonomy of East Asian freshwater mytiloids and provides new insights into marine–freshwater transitions and the evolutionary significance of ancient lake systems.

## Introduction

Freshwater ecosystems have repeatedly served as important arenas for evolutionary diversification within primarily marine bivalve lineages (Bogan 2008; Graf 2013). Among these, Mytiloidea is overwhelmingly marine, with only a few lineages successfully colonising freshwater environments. Such ecological transitions are rare and evolutionarily significant, often accompanied by substantial morphological modification and convergence, which may obscure phylogenetic relationships within the Mytiloidea (Sokolova et al. 2021; Tan and Tan 2024). Recent phylogenetic studies have further suggested that Mytiloidea comprises multiple distinct clades, with some freshwater-associated taxa, including *Sinomytilus* and *Limnoperna*, placed within Modiolidae (Tan and Tan 2024).

The genus *Sinomytilus* Thiele, 1934 was established based on material from Southeast Asia and represents one of the few freshwater mytiloid lineages (Morton and Dinesen 2010; Graf 2013). Despite its early description, the genus remains poorly understood. In China, *Sinomytilus* has long been represented solely by *Sinomytilus swinhoei* (H. Adams, 1870) from the Yangtze River system (Huber 2010; Morton and Dinesen 2010; Wang et al. 2024). Recent surveys have reported additional freshwater mytiloid taxa from southwestern China, including a small-bodied species from Lake Fuxian provisionally assigned to *Sinomytilus* based on shell morphology and limited molecular data (Wang et al. 2024). However, the diagnostic boundaries of *Sinomytilus* have never been rigorously tested, and the relationships among species currently placed in this genus remain unclear.

In addition to uncertainties at the generic level, phylogenetic relationships within freshwater mytiloid bivalves remain problematic at the species level. Previous molecular studies have yielded conflicting results regarding the monophyly of *Limnoperna*: while Tan and Tan (2024) suggested that the genus is not monophyletic, with the type species *L. siamensis* and the widely invasive *L. fortunei* failing to form a consistent clade, a recent analysis by Bolotov et al. (2026) recovered these species as a clade. This discordance highlights the need for further phylogenetic assessment of the genus using expanded taxon and marker sampling. Furthermore, discrepancies between molecular clock estimates and concatenated phylogenetic analyses have resulted in conflicting evolutionary scenarios for these taxa (Sokolova et al. 2021; Tan and Tan 2024). These inconsistencies highlight the limitations of single-gene or sparsely sampled datasets and underscore the necessity of more comprehensive multi-locus approaches.

Shell morphology alone may be insufficient for defining generic boundaries in freshwater mytiloid bivalves, as similar shell forms can arise independently under comparable ecological pressures, a pattern widely documented in molluscs (Chen et al. 2025c; Zhang and Wang 2025). Morphological convergence associated with freshwater adaptation is particularly widespread in Mytiloidea, further limiting the diagnostic value of shell characters for higher-level taxonomy (Morton and Dinesen 2010; Morton 2015; Morton et al. 2020; Sokolova et al. 2021; Tan and Tan 2024). Consequently, an integrative approach combining morphology, anatomy, and multi-locus molecular phylogenetics is essential for resolving generic boundaries and evolutionary relationships within freshwater modiolids.

Southwestern China provides an ideal natural setting for such integrative investigations (Chen et al. 2025a, 2025b, 2025c; Zhang et al. 2025; Zheng et al. 2026a). The region encompasses both ancient lakes and palaeolake systems that collectively preserve a wide temporal spectrum of freshwater faunas. Lake Fuxian, one of the deepest lakes in the Yunnan–Guizhou Plateau, is characterised by relatively stable environmental conditions and harbours a number of endemic aquatic taxa (Cui et al. 2008; Wang et al. 2024; Zhang et al. 2024; Chen et al. 2025b; He et al. 2025; Zheng et al. 2026b). In contrast, the Luliang Basin once comprised a group of interconnected Quaternary lakes that were artificially drained during the mid-20^th^ century, preserving a rich subfossil molluscan assemblage, including Viviparidae and Pomatiopsidae (Ji 1989; Zhang and Wang 2023; Xiang et al. 2025, 2026). These contrasting temporal and environmental contexts provide a natural framework for investigating the diversification and persistence of freshwater mytiloid lineages.

In addition to lacustrine material from southwestern China, bivalves traditionally assigned to *Sinomytilus* are also distributed in major river systems (Adams 1870; Morton and Dinesen 2010; Graf 2013). However, the taxonomic status and evolutionary relationships of these populations have not yet been rigorously evaluated using integrative approaches.

In the present study, we examine both extant and subfossil material referable to *Sinomytilussensu lato* from multiple geographic and temporal contexts, including Lake Fuxian, the Luliang palaeolake basin, and major river systems in China. Based on detailed comparisons of shell morphology, anatomical observations, and multi-gene phylogenetic analyses using five genetic markers (COI, 16S rRNA, 18S rRNA, 28S rRNA, and H3), we show that *Sinomytilus* as currently defined is not monophyletic. In addition, our results recover *Limnoperna siamensis* and *L. fortunei* as a well-supported monophyletic lineage. Accordingly, we revise the taxonomy of *Sinomytilussensu lato* and establish a new genus: *Shenzhousia* gen. nov., with *Sinomytilus swinhoei* as the type species. In addition, we describe a new subfossil species, *Shanomytilus frendae* sp. nov., from the Quaternary lacustrine deposits of the Luliang Basin.

These findings provide new insights into the systematics of freshwater mytiloid bivalves in East Asia and contribute to a better understanding of evolutionary transitions between marine and freshwater environments, as well as the role of ancient and vanished lake systems in shaping regional biodiversity.

## Materials and methods

### Taxon sampling

**Figure 1.**
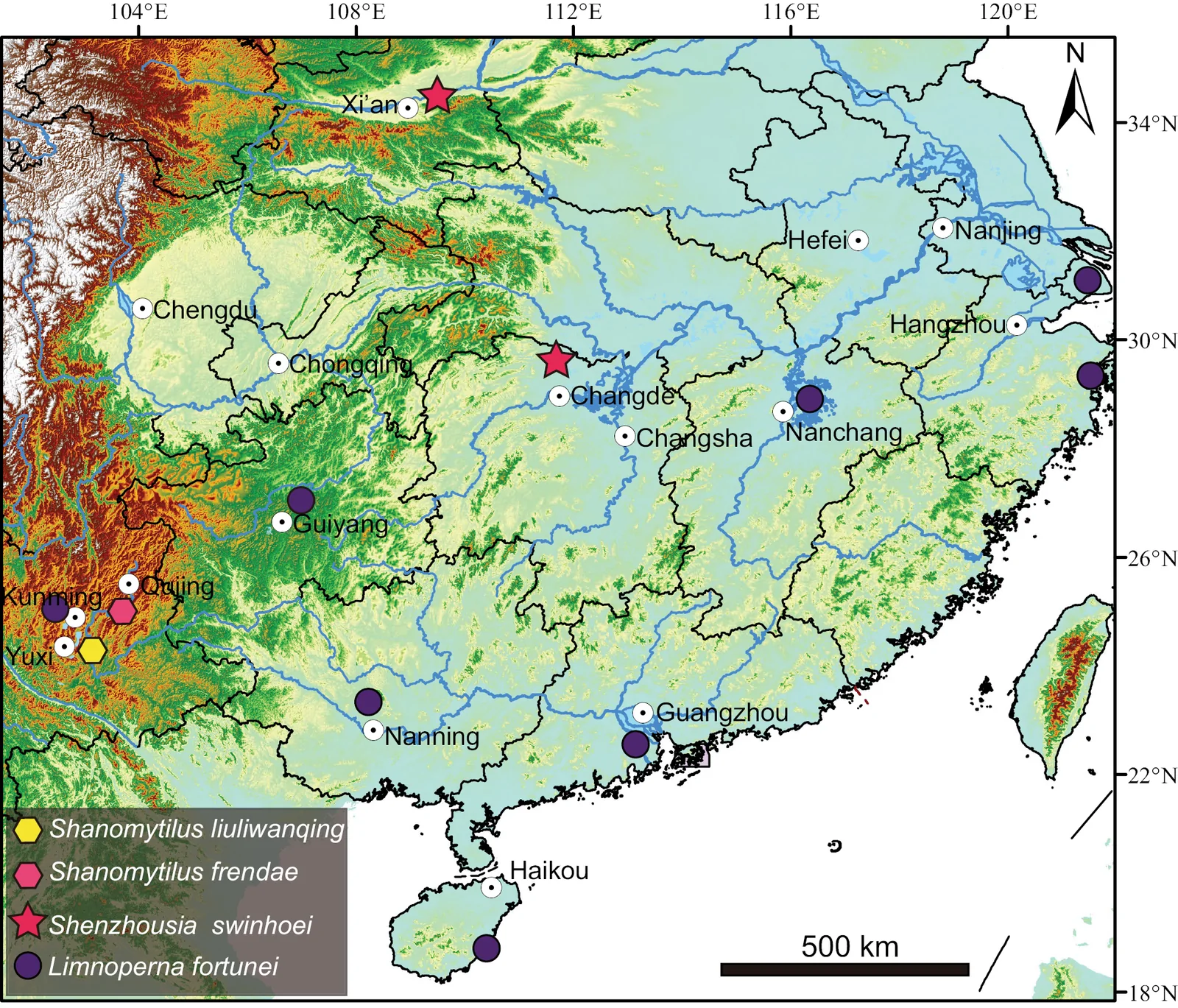
Map showing the sampling localities of specimens examined in this study from China.

*Shenzhousia swinhoei* (formerly *Sinomytilus swinhoei*) were collected from the Yangtze River system in Changde, Hunan Province (August 2025), and from the Yellow River system in Xi’an, Shaanxi Province (May 2025). *Limnoperna fortunei* was collected from the following localities: Jiangmen, Guangdong Province (March 2026); Minhang District, Shanghai (March 2026); Poyang Lake, Nanchang, Jiangxi Province (January 2024); Ninghai, Ningbo, Zhejiang Province (January 2026); Kaiyang, Guiyang, Guizhou Province (February 2026); Nanning, Guangxi Province (March 2026); Kunming, Yunnan Province (March 2026); and Haikou, Hainan Province (March 2025). *Shanomytilus liuliwanqing* was collected from Lake Fuxian, Yunnan Province (January 2023 and July 2025). Subfossil specimens (*Shanomytilus frendae*) were collected from the Luliang Basin, Yunnan Province (February 2024) (Fig. 1). Specimens were preserved either in 95% ethanol or as dry shells, and were deposited at the Zoological Museum of Capital Normal University, Beijing, China (CNU) and the Museum of Biology, Nanchang University, China (NCUMB). The shell characteristics were measured to the nearest 0.1 mm using digital calipers for each individual. Photographs were taken with a Sony A7CII. Prior to scanning electron microscopy (SEM), the shells were cleaned and air-dried in a clean environment for eight hours, after which their surfaces were sputter-coated with gold. Subsequently, the samples were subjected to examination via SEM (ZEISS G300).

### DNA extraction, PCR amplification, and mitogenome assembly

Total genomic DNA was isolated from a small piece of foot tissue of each ethanol-preserved specimen using a Trelief™ Animal Genomic DNA Kit (Tsingke®). Partial sequences of COI, 16S, 18S, 28S, and H3 were amplified using the primers and PCR conditions following Liu et al. (2018). Each PCR reaction was performed in a total volume of 20 μL, including 9 μL of Taq Plus Master Mix II (Vazyme Biotech Co., Ltd., Nanjing, China; Cat# P213-01), 8 μL of double distilled water, 1 μL of each primer and 1 μL of the DNA template. Sequences were obtained by bidirectional automated sequencing using forward and reverse primers on the Applied Biosystems 3730 platform (Sangon Biotech, Shanghai, China). Qualified genomic DNA samples were dispatched to Novogene (Beijing, China) for library construction and sequencing. After discarding low-quality reads, clean reads were obtained and assembled *de novo* by CLC Genomic Workbench (v. 12.0; see https://digitalinsights.qiagen.com/). Contigs identified as mitogenome sequences were checked manually for overlap at the beginning and end, resulting in a circular mitogenome. The newly produced mitogenome was checked manually and annotated with Geneious (v. 11.0; see https://www.geneious.com; Kearse et al. 2012). The initial annotation of the mitogenome was carried out using the MITOS web server (see http://mitos.bioinf.uni-leipzig.de/index.py; Bernt et al. 2013). ARWEN (see http://130.235.244.92/ARWEN/; Laslett and Canbäck 2008) was also used to identify the locations of all tRNA genes. The annotations for two rRNA genes were further refined based on the positions of neighbouring genes. Open Reading Frame Finder (ORF Finder, see http://www.ncbi.nlm.nih.gov/orffinder/) and BLAST searches (see http://blast.ncbi.nlm.nih.gov/) were used to identify 13 protein-coding genes (PCGs).

### Alignments and partitioning strategies

Three datasets were constructed in this study: (1) a mitochondrial dataset (containing COI and 16S) (Suppl. material 1) (2) a multilocus dataset (containing COI, 16S, 18S, 28S and H3) (Suppl. material 2) and (3) mitogenomic dataset (including 12 PCGs excluding ATP8) (Suppl. material 3); Sequences were aligned using MAFFT v. 7.505 based on the L-INS-i method (Katoh and Toh 2008). In addition, all PCGs were translated into amino acid sequences with MEGA X (v. 10.2; Kumar et al. 2018) to detect the existence of stop codons and sequencing errors. No such stop codons were detected, confirming the absence of sequencing errors and pseudogenes. Subsequently, the PCGs were codon-aligned by the program MUSCLE (v. 5; Edgar 2004). Pairwise distances between species were also calculated using MEGA X (Kumar et al. 2018). Gblocks (ver. 0.91b; Castresana 2000) was used to exclude ambiguous regions from the alignment of each gene. We used PartitionFinder 2 (v. 2.3.4; Lanfear et al. 2017) to select the best-fit partitioning schemes and models under greedy search with unlinked branch lengths. Predefined data blocks for partitioning scheme searches were designated by gene region (rRNA gene) or codon position (PCG). Branch lengths were permitted to be unlinked, with the AICc method utilised to determine model selection and partitioning schemes (Suppl. material 4).

### Phylogenetic analyses

Maximum likelihood (ML) analyses were conducted using the ML + fast bootstrap approach and 1000 replicates in raxmlGUI (v. 2.0, Edler et al. 2020). The Bayesian inference (BI) analysis was carried out by running two runs of 10 million generations of Metropolis-coupled Markov chain-Monte Carlo (MCMC) in MrBayes (v. 3.2.6, Ronquist et al. 2012). Each run used a random tree as a starting tree and collected a sampling tree every 1000^th^ generation with a 25% burn-in. Sufficient chain mixing was deemed achieved when the mean standard deviation of split frequencies was below 0.01 and the potential scale reduction factor (PSRF) was 1.

### Divergence time estimation

Divergence times were estimated using BEAST 2 (v. 2.6.7; Bouckaert et al. 2019) based on the concatenated dataset of five molecular markers. Substitution models assigned to each partition are consistent across the phylogenetic analysis (Suppl. material 4). An uncorrelated lognormal relaxed molecular clock model was employed and the Yule speciation process was selected as the tree prior. Three fossil calibration points were implemented as normal distribution priors on internal nodes. The root of Mytilidae was constrained to 419–427 Mya (mean = 423 Mya, SD = 2.2) based on the earliest mytilid fossils *Phthonia regularis* and *Mytilus* sp. from the Silurian (Berry and Boucot 1973; Kříž 2008). The node of *Vulcanidas insolatus* was calibrated using the Middle Eocene fossil *Vulcanidas goederti* (Kiel and Amano 2013) with a normal prior spanning 43.5–48.5 Mya (mean = 46 Mya, SD = 1.5). The terminal node of *Musculista senhousia* was constrained by a Pleistocene fossil of the same species (Okamoto and Ibaraki 1988) with a normal prior of 0.78–2.59 Mya (mean = 1.68 Mya, SD = 0.55).

Four independent MCMC runs were executed for 100 million generations each, sampling every 10,000 generations, with substitution and clock models unlinked between partitions. Convergence and effective sample size (>200) of parameters were assessed in Tracer. LogCombiner v.2.6.7 was used to combine trees from the four runs, discarding the first 10% of generations as burn-in. A maximum clade-credibility tree was processed from 36,004 trees in TreeAnnotator v.2.6.7 with the posterior probability limit set to 0.5 and mean node heights summarised. The maximum clade credibility (MCC) tree was extracted in the TreeAnnotator software of the BEAST package.

## Results

### Phylogenetic analyses

**Figure 2.**
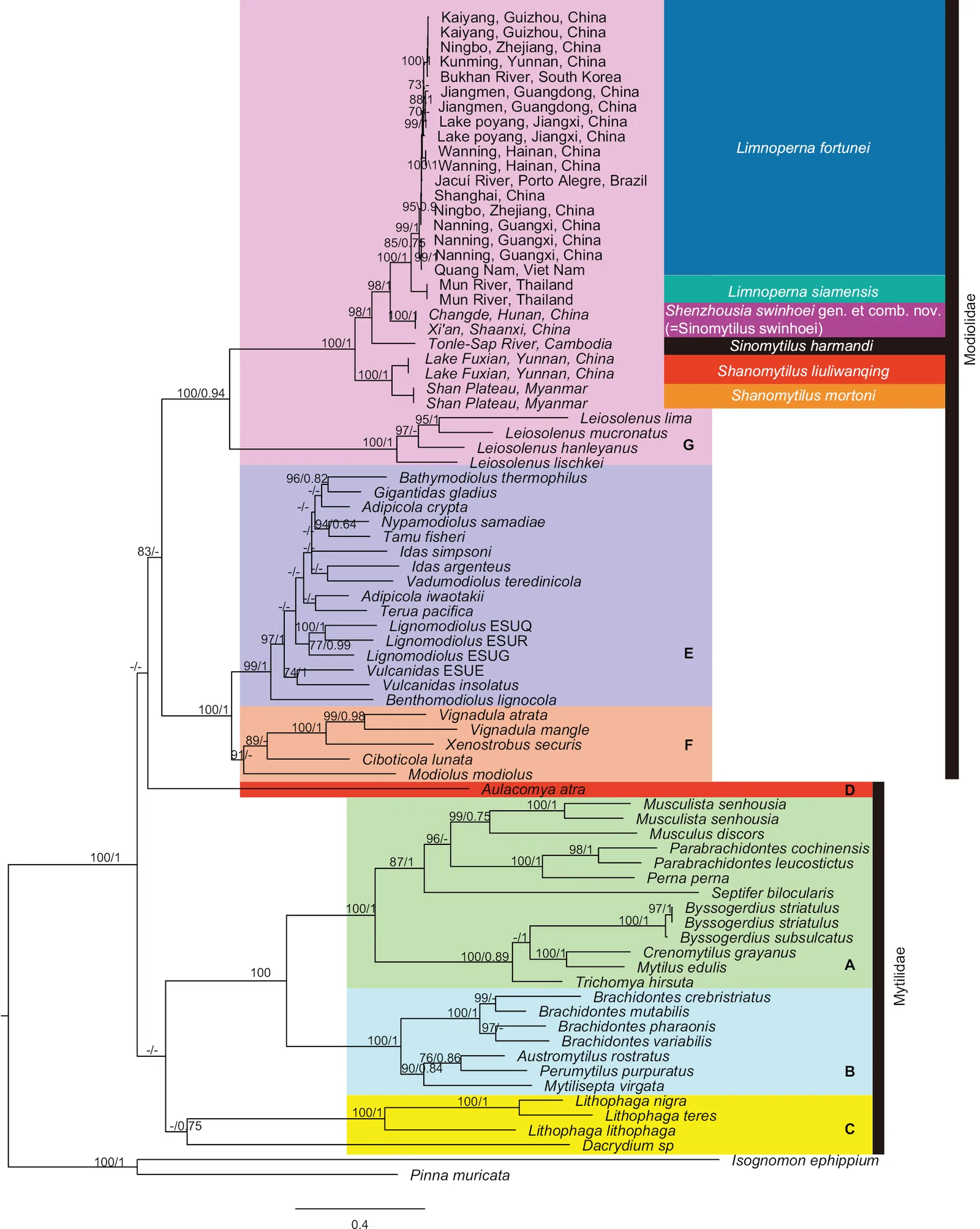
Maximum likelihood (ML) tree reconstructed from the mitochondrial dataset (COI, 16S). Values at nodes indicate bootstrap support (BS) and Bayesian posterior probabilities (PP), respectively.

**Figure 3.**
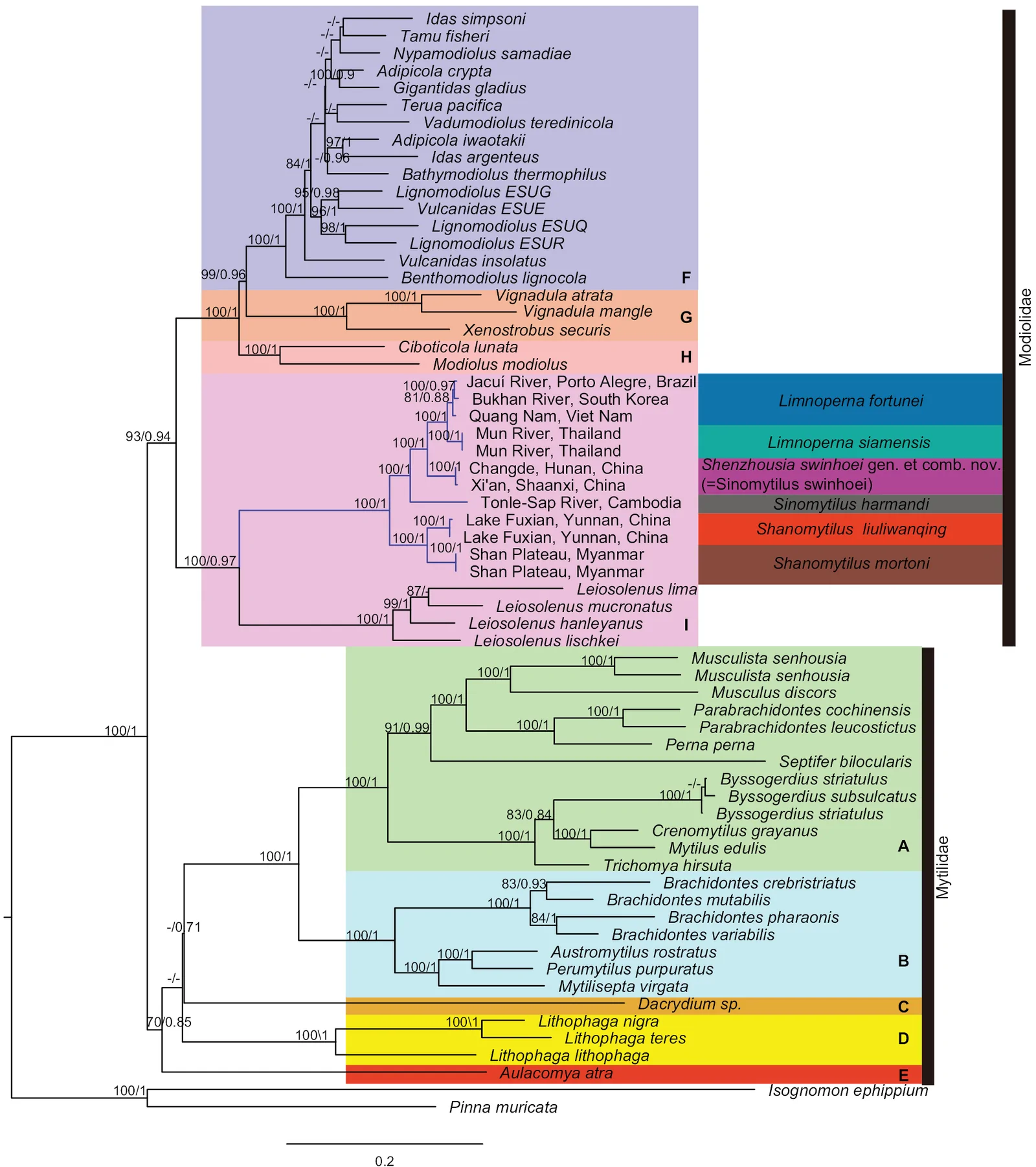
Maximum likelihood (ML) tree reconstructed from the multilocus dataset (COI, 16S, 18S, 28S and H3). Values at nodes indicate bootstrap support (BS) and Bayesian posterior probabilities (PP), respectively.

The topological structures of the resulting trees from maximum likelihood (ML) and Bayesian inference (BI) analyses were largely consistent for both the mitochondrial and multilocus datasets (Figs 2, 3).

For the mitochondrial dataset, two major clades were recovered, Clade I (Mytilidae, excluding *Aulacomya atra*) and Clade II (Modiolidae), although with relatively low support values. Within Clade I, three subclades (A–C) were resolved, while Clade II comprised four subclades (D–G), also with generally low support. Within Clade II (Modiolidae), specimens of *Limnoperna fortunei* collected from various localities across China, as well as from Porto Alegre (Brazil), Quang Nam (Viet Nam) and the Bukhan River (South Korea), formed a well-supported monophyletic group (100%/1). The broader freshwater lineage, including all freshwater mytiloid taxa, also formed a well-supported monophyletic clade (100%/1). Within this lineage, *L. fortunei* and *L. siamensis* formed a monophyletic clade (100%/1). Species traditionally assigned to *Sinomytilus* were not recovered as monophyletic. Instead, *Sinomytilus swinhoei* clustered with *Limnoperna* species with strong support (98%/1). This clade then grouped with *Sinomytilus harmandi* (98%/1), followed by *Shanomytilus liuliwanqing* and *S. mortoni* (100%/1), collectively forming the freshwater lineage. This entire freshwater clade was sister to the genus *Leiosolenus* (100%/0.94) (Fig. 2).

For the multilocus dataset, two major clades were also recovered, Clade I (Mytilidae) and Clade II (Modiolidae), each forming a monophyletic group with support values of 70%/0.85 and 93%/0.94, respectively. Within Clade I, five subclades (A–E) were resolved, whereas Clade II comprised four subclades (F–I). Within Clade II (Modiolidae), the freshwater lineage formed a well-supported monophyletic group (100%/0.99). Within this lineage, *L. fortunei* and *L. siamensis* formed a monophyletic clade (100%/1). Species traditionally assigned to *Sinomytilus* were not recovered as monophyletic. Instead, *Sinomytilus swinhoei* clustered with *Limnoperna* species with strong support (100%/1). This clade then grouped with *Sinomytilus harmandi* (100%/1), followed by *Shanomytilus liuliwanqing* and *S. mortoni* (100%/1), collectively forming the freshwater lineage. This entire freshwater clade was sister to the genus *Leiosolenus* (100%/0.99) (Fig. 3).

**Figure 4.**
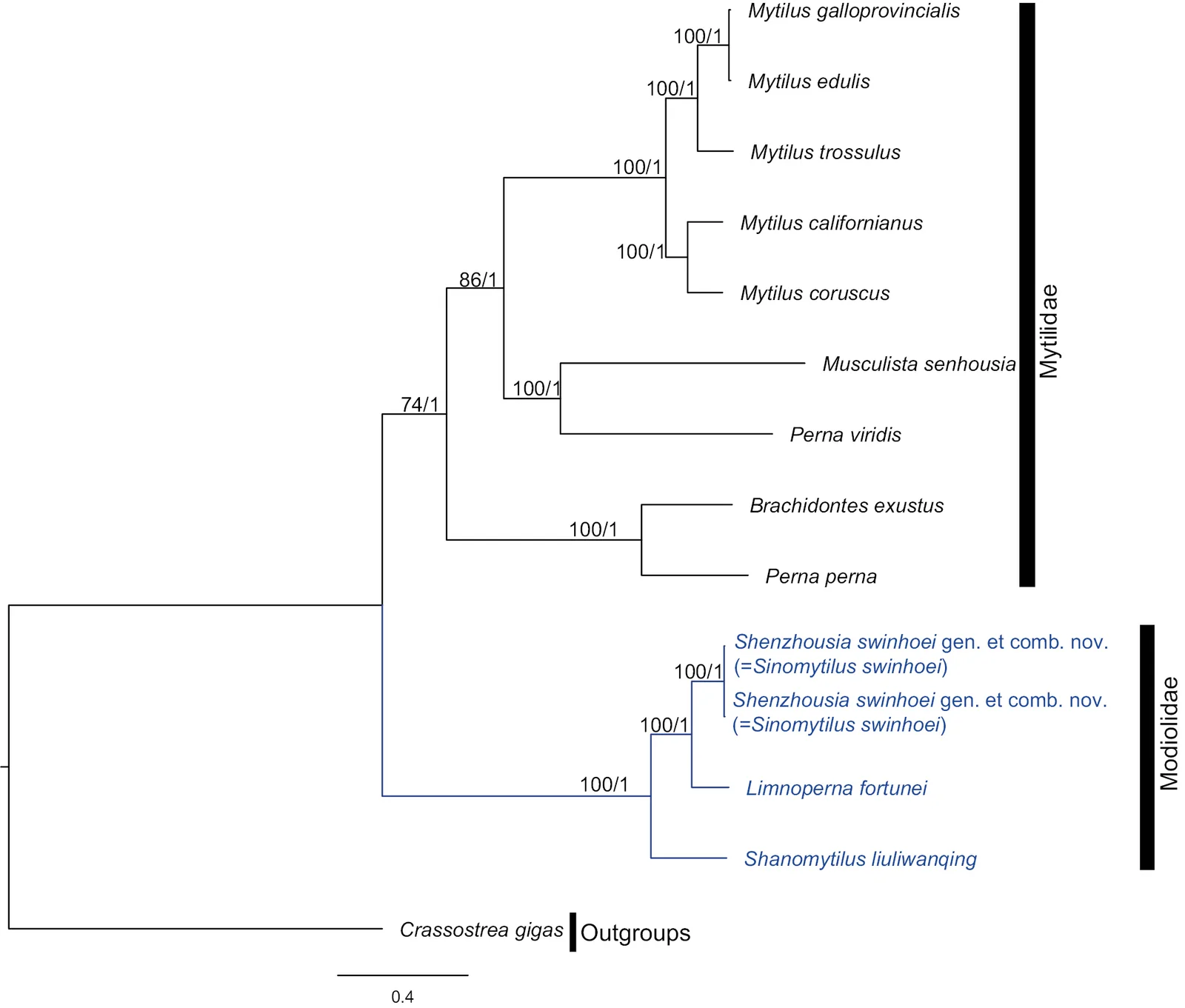
Maximum likelihood (ML) tree reconstructed from 12 protein-coding genes (excluding ATP8) of the mitochondrial genome. Support values at nodes are bootstrap (BS) / Bayesian posterior probabilities (PP).

The mitogenome-based phylogenetic tree, comprising eight genera, also recovered Mytilidae and Modiolidae as monophyletic groups, with support values of 74%/1 and 100%/1, respectively. Within this tree, *Sinomytilus swinhoei* clustered with *Limnoperna fortunei* (100%/1), and this clade was sister to *Shanomytilus liuliwanqing* with strong support (100%/1). The genus *Sinomytilussensu lato* was found to be polyphyletic (Fig. 4).

Therefore, one new generic placement is proposed: *Shenzhousia swinhoei* gen. et comb. nov. (previously *Sinomytilus swinhoei*). Based on COI gene sequences, the mean uncorrected p-distance between *Limnoperna fortunei* and *L. siamensis* was 6.67%. The mean uncorrected p-distance between *Shanomytilus liuliwanqing* and *S. mortoni* was 7.53%. The distance between *Sinomytilus harmandi* and *Shenzhousia swinhoei* was 14.9%, and between *Sinomytilus harmandi* and *Shanomytilus* species ranged from 15.6% to 16.2%. The distances between *Sinomytilus harmandi* and *Limnoperna* species ranged from 13.8% to 14.7%.

### Time-calibrated species tree

**Figure 5.**
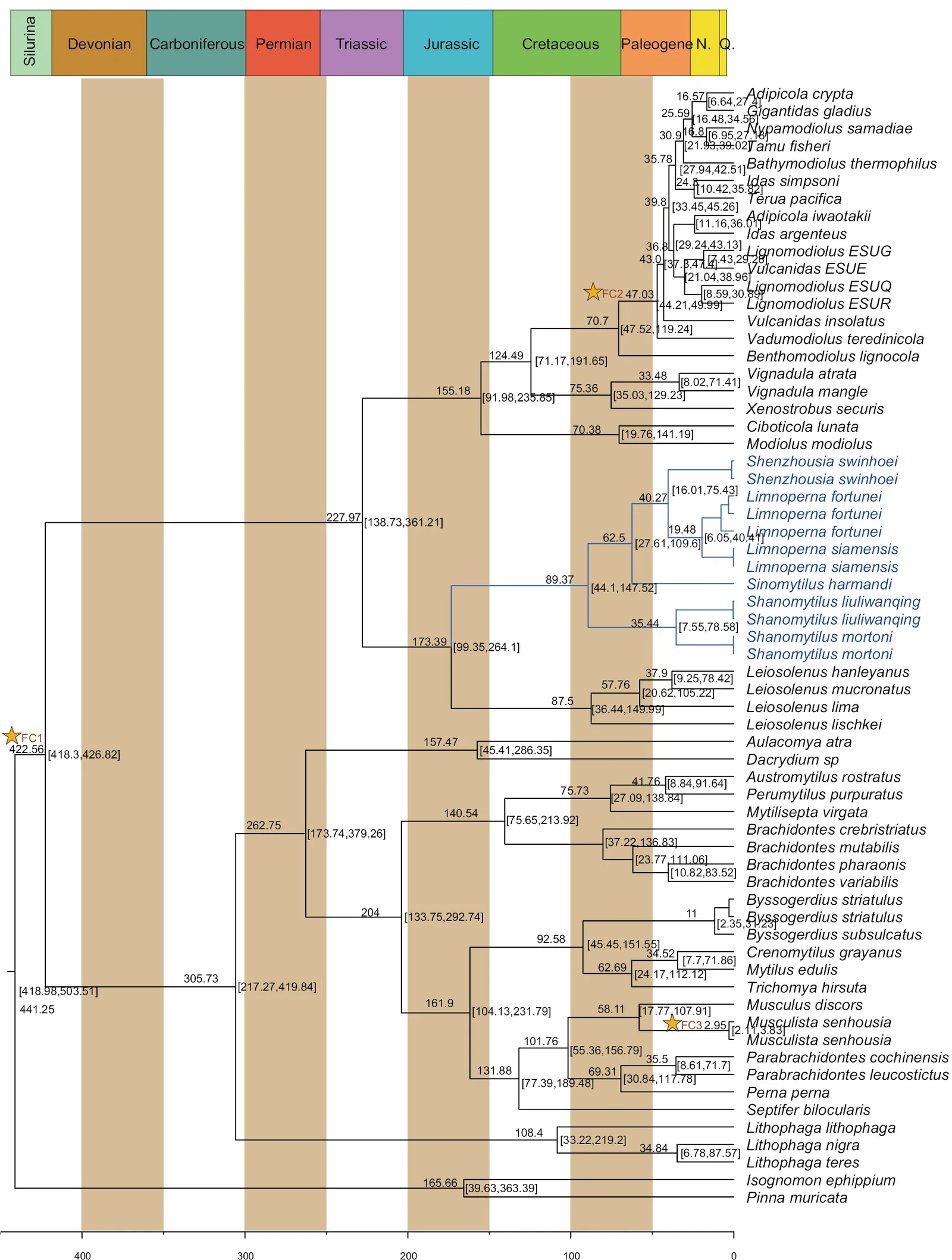
Maximum clade credibility tree based on five molecular markers (COI, 16S, 18S, 28S, and H3) obtained from BEAST. Calibration points are marked as FC1, FC2, and FC3. The freshwater lineage is highlighted in blue. Numbers above the branches represent the mean age for the node directly below the branch. Numbers in brackets represent the 95% confidence interval for the ages of the nodes.

Divergence times were estimated using BEAST2 based on the five-gene dataset (COI, 16S, 18S, 28S, H3) under an uncorrelated lognormal relaxed clock model with a calibrated Yule tree prior. Our time-calibrated phylogeny (Fig. 5) revealed that the freshwater lineage (comprising *Shenzhousia swinhoei*, *Limnoperna fortunei*, *L. siamensis*, *Sinomytilus harmandi*, *Shanomytilus liuliwanqing* and *S. mortoni*) diverged from the genus *Leiosolenus* at approximately 173.39 Ma (95% HPD: 99.35–264.1 Ma). Although the 95% HPD intervals for these deep nodes were relatively wide, likely reflecting limited fossil calibrations within Mytiloidea, the median estimates provide a useful temporal framework.

Within the freshwater lineage, successive divergence events were estimated. *Shanomytilus* split from the remaining taxa at ~89.37 Ma (95% HPD: 44.1–147.52 Ma). *Sinomytilus harmandi* subsequently diverged from the clade containing *Shenzhousia swinhoei* and the two *Limnoperna* species at ~62.5 Ma (95% HPD: 27.61–109.6). Finally, *Shenzhousia swinhoei* diverged from *Limnoperna fortunei* and *L. siamensis* at ~40.27 Ma (95% HPD: 16.01–75.43 Ma). Most generic-level divergences within Modiolidae and Mytilidae occurred during the Late Cretaceous.

Collectively, these estimates suggest that the freshwater mytiloid lineage originated in the Middle Jurassic, with subsequent cryptic diversification during the Cretaceous and more pronounced lineage accumulation during the Late Cretaceous to Early Paleogene.

### Systematic account

**Superfamily Mytiloidea Rafinesque, 1815**

**Family Modiolidae G. Termier & H. Termier, 1950**

**Subfamily Limnoperninae Scarlato & Starobogatov, 1979**

#### Genus Shanomytilus

Taxon classificationAnimaliaMytilidaModiolidae

Bolotov, Aksenova, Bespalaya & Kondakov, 2026

DD0AEC9E-0D93-581D-8CC7-F017B10CD136

##### Type species.

*Shanomytilus mortoni* Bolotov, Aksenova, Bespalaya & Kondakov, 2026 (by original designation).

#### Shanomytilus frendae

Taxon classificationAnimaliaMytilidaModiolidae

Wang, Zheng, He & Xiang sp. nov.

779FA7C6-F124-55DF-96D6-3527F46012ED

https://zoobank.org/D54BA983-CEA1-404C-8BB4-05658C8CB006

##### Material examined.

**Figure 6.**
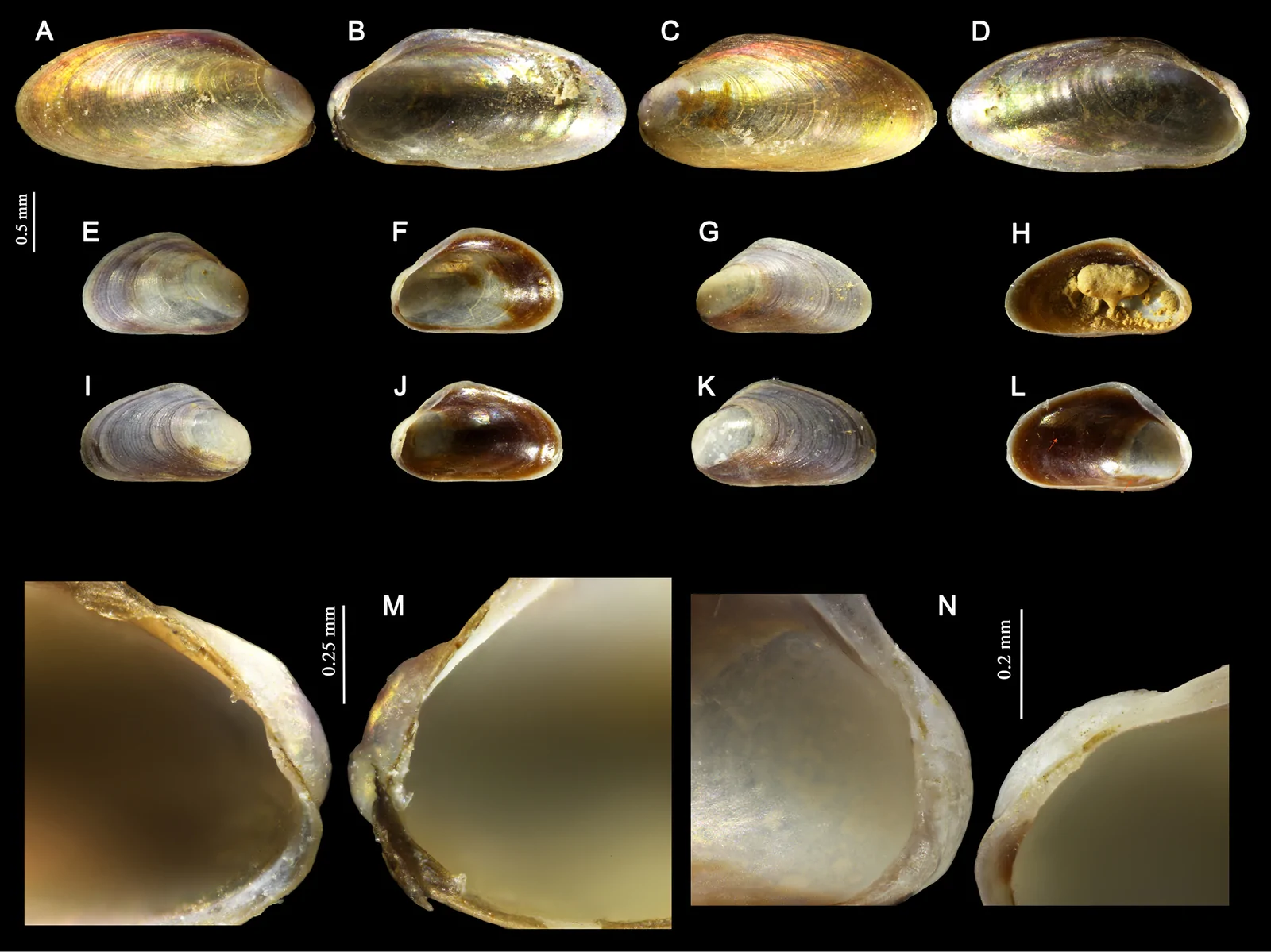
Species of *Shanomytilus*. **A–D**. *S. liuliwanqing*, CNU.Mol.2617; **E, F**. *S. frendae*, holotype, CNU.Mol.2606; **G–L**. *S. frendae*, paratypes, CNU.Mol.2607–2609; **M**. Anterior region of *S. liuliwanqing*; **N**. Anterior region of *S. frendae*. Red triangles indicate the posterior adductor muscle scar and anterior adductor muscle scar.

***Holotype***. • CNU.Mol.2606, shell length 1.39 mm, width 0.92 mm, Wanghai Temple, buried in soil, Luliang County, Yunnan Province, China, 25°01'14"N, 103°40'11"E, February 2024 collected by Xing-Xiao Wang. ***Paratypes***. • 9 specimens. CNU.Mol.2607-CNU.Mol.2615, shell length 1.42–1.55 mm, width 0.83–0.98 mm, Locality and habitat same as holotype (Fig. 6E–L).

##### Diagnosis.

Shell minute, thin, translucent and fragile, ovately triangular; posterior margin broadly rounded, anterior end slightly narrowed; umbo low, subterminal; shell surface smooth, with fine concentric growth lines only.

##### Description.

Shell minute (length 1.39–1.55 mm), thin and fragile, outline ovately triangular to subovate, moderately inflated. Umbo subterminal, low but distinct. Posterior margin broadly rounded; anterior margin gently tapering and narrowly rounded. Dorsal margin nearly straight, forming an obtuse angle of ~120° with the posterior margin; ventral margin nearly straight, very slightly concave just posterior to mid-length.

Shell surface smooth and glossy, bearing fine, closely spaced concentric growth lines; radial sculpture absent. Shell margin whitish and slightly thickened.

Interior smooth, nacre thin and weakly iridescent, pale silvery to light brown; anterior adductor muscle scar and pallial line shallow; posterior adductor muscle scar subovate, very shallow. Hinge edentulous. Ligament relatively long and narrow, occupying ~36–39% of the shell length (Fig. 6E–L).

##### Habitat and distribution.

This species is currently known only from lacustrine deposits at the type locality near the downtown area of Luliang County. All examined material consists of disarticulated valves, and no complete articulated specimens have been recovered. The Mollusca from the same locality also include *Yinli zhuxiangyueae* Y.-M. He, H.-Q. Xiang, H. Gao & H. Chen, 2025 (Gastropoda: Pomatiopsidae), *Tchangmargarya cuan* L.-J. Zhang & X.-D. Wang, 2023 (Gastropoda: Viviparidae), as well as several other unidentified species of Pomatiopsidae and Squamapicidae.

##### Etymology.

The specific name frendae is named after Frenda Seivelun, a character from the Japanese light novel series A Certain Magical Index and its spin-off A Certain Scientific Railgun. The name refers to the peculiar preservation of the species, which is known only from isolated valves rather than articulated shells, and alludes to the character’s well-known fate. We suggest the Chinese common name to be ‘芙兰达掸贻贝’. Gender: feminine.

##### Remarks.

**Figure 7.**
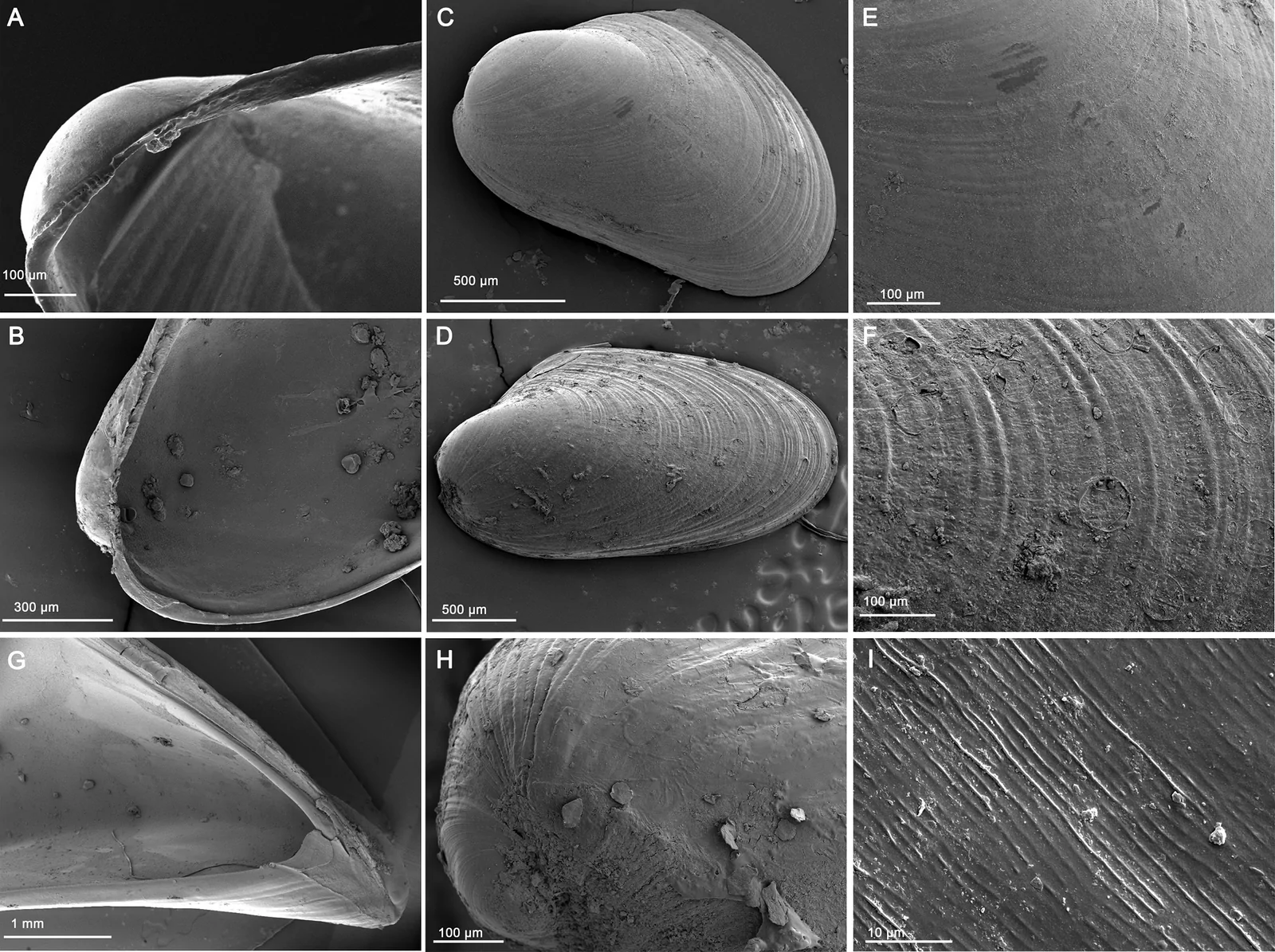
Scanning electron microscope photograph of *Shanomytilus* and *Shenzhousia* species. **A, B**. Anterior region of *S. frendae* and *S. liuliwanqing*; **C, D**. External view of *S. frendae* and *S. liuliwanqing*; **E, F**. Outer surface of *S. frendae* and *S. liuliwanqing*; **G**. Internal view of *Shenzhousia swinhoei*; **H**. Umbo of *S. swinhoei*; **I**. Ultrastructure of the outer surface of *S. swinhoei*.

*Shanomytilus frendae* sp. nov. conforms well to the diagnostic conchological features of *Shanomytilus*, particularly in having a minute, thin-walled shell with subterminal umbones, an edentulous hinge, absence of an internal anterior septum (Figs 6N, 7A, 7C), and a shell surface bearing concentric growth lines without radial sculpture (Fig. 7E). It can be readily distinguished from the type species, *S. mortoni*, by its substantially smaller shell size (SL 1.39–1.55 mm vs 3.31–5.69 mm in the examined paratypes of *S. mortoni*) and its more compact shell profile. *Shanomytilus frendae* is relatively short and high, with an ovately triangular outline and a shortened posterior region, whereas *S. mortoni* is more elongate and modioliform. The transition between the dorsal and posterior margins is also distinctly more angular in *S. frendae*, forming a relatively acute angle, whereas in *S. mortoni* the two margins meet at a broader, more rounded angle, contributing to its more smoothly elongate posterior profile. Moreover, *S. mortoni* possesses distinctly convex umbones and a prominent angled or rounded umbonal keel, whereas the umbones and umbonal keel are less strongly developed in *S. frendae*. The ventral margin of *S. frendae* is nearly straight, whereas that of *S. mortoni* may be straight to concave.

The new species is also readily distinguished from *S. liuliwanqing* by its markedly smaller shell size (SL 1.39–1.55 mm vs 2.51–3.23 mm in *S. liuliwanqing*) (Fig. 6A–L). It further differs in shell proportions and outline, *S. frendae* being more compact and ovately triangular, with a relatively shortened posterior region. The dorsal and posterior margins meet at an obtuse angle of ~120°, compared with ~150° in *S. liuliwanqing*, and the ventral margin is straighter in *S. frendae*. Consequently, *S. frendae* has a relatively shorter and higher shell profile, whereas *S. liuliwanqing* is more elongate, with a more extended posteroventral region (Fig. 6M, N). The ligament is also proportionally longer in *S. frendae*, occupying ~36–39% of shell length, compared with 21–25% in *S. liuliwanqing*. This difference suggests a divergent allometric relationship between ligament length and shell size between the two species. Furthermore, observations under scanning electron microscopy (SEM) showed that the concentric growth lines of the new species are comparatively smoother and less distinctly expressed, whereas in *S. liuliwanqing* they are more prominent and sharply defined (Fig. 7C–F). However, all available specimens of *S. frendae* are subfossil (tentatively dated to the Quaternary), and the comparatively weak concentric growth lines and porous appearance of the shell surface may be attributable, at least in part, to long-term burial and post-depositional alteration rather than representing intrinsic diagnostic characters. Prolonged diagenetic processes, including surface dissolution and recrystallisation, may have modified the original shell microstructure and sculpture (Janiszewska et al. 2018; Forjanes et al. 2022). These surface features are therefore treated with caution and are not considered primary diagnostic characters of the new species.

#### Shenzhousia

Taxon classificationAnimaliaMeganisopteraMeganeuridae

Zheng, Wang, He & Xiang gen. nov.

4302FF42-B3BA-5A74-A919-50CCA6694FCD

https://zoobank.org/6AB550AF-1AC1-4381-9430-6C0EBC0A5A14

##### Type species.

*Dreissena swinhoei* H. Adams, 1870.

##### Diagnosis.

**Figure 8.**
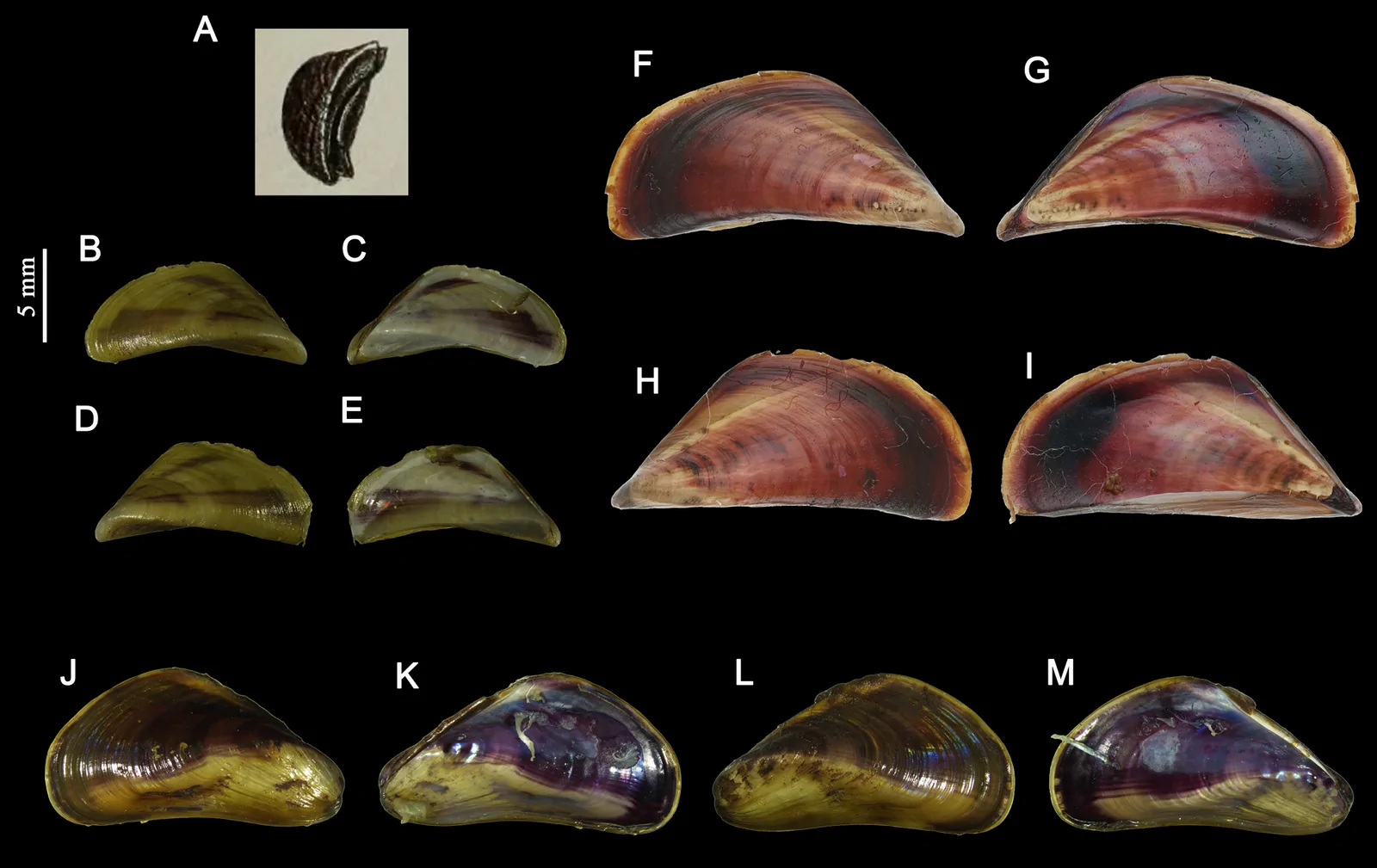
Species of *Shenzhousia* (A–E), *Sinomytilus* (F–I) and *Limnoperna* (J–M). **A**. Original figure of *Dreissena swinhoei* from Adams (1870: pl. 27, fig. 13); **B–E**. *Shenzhousia swinhoei*, neotype, NCUMB.CDSS2601 collected from Changde, Hunan, China; **F–I**. *Sinomytilus harmandi*, collected from Tonle Sap Lake, Cambodia, CNU.Mol.2616; **J–M**. *Limnoperna fortunei*, collected from Jiangmen, Guangdong, China, NCUMB.JMLF01. Red triangles indicate the posterior adductor muscle scar.

Shell medium-sized, solid, strongly inequilateral and heteromyarian, characteristically axe-shaped, elongate wedge-shaped to narrowly triangular. Umbo terminal to subterminal, prominent. Anterior end sharply tapering; posterior margin broadly rounded. Ventral margin distinctly concave. A single continuous ridge extending from the umbo to the posteroventral extremity. Shell surface smooth, with fine concentric growth lines and slight longitudinal striations under SEM; radial sculpture absent. Hinge edentulous; ligament narrow and externally inconspicuous. Interior smooth, weakly nacreous; anterior adductor muscle scar absent; posterior adductor muscle scar shallow and subovate. Septum weak and inconspicuous. Anterior adductor muscle strongly reduced (Fig. 8A–E).

##### Etymology.

The generic name *Shenzhousia* is derived from “Shenzhou”, a traditional literary term referring to China. The name refers to the currently known distribution of the species included in this genus, which are presently recorded from the Yangtze River and Yellow River basins. We suggest the Chinese common name to be ‘神州贝’. Gender: feminine.

##### Remarks.

*Shenzhousia* resembles *Sinomytilus* Thiele, 1934 and *Limnoperna* Rochebrune, 1882 in possessing an edentulous hinge and a generalised freshwater mytilid shell form. However, it differs from both genera in its distinctly axe-shaped shell, with a strongly developed continuous keel extending from the umbo to the posteroventral extremity, a sharply tapering anterior end, and a prominently elevated, acute, and slightly sinuous umbonal region.

It differs from *Sinomytilus* in shell microsculpture: under SEM, the shell surface of *Shenzhousia* shows slight longitudinal striations (Fig. 7I), whereas such striations are not evident in *Sinomytilus*, whose shell surface instead bears conspicuous pores in fast-growth bands (Fig. 7G–I; Morton and Dinesen 2010).

*Shenzhousia* differs from *Limnoperna* in its more strongly inequilateral and axe-shaped shell, the distinctly concave ventral margin, the prominent continuous ridge, and the presence of a residual septum. Furthermore, *Limnoperna* possesses a well-developed anterior adductor scar and lacks any septum, whereas in *Shenzhousia* the anterior adductor is strongly reduced and the septum remains as a vestigial structure (Fig. 8J–M). Although slight longitudinal striations are also present under SEM in *Limnoperna*, the overall shell form of *Shenzhousia* is markedly more specialised (Morton and Dinesen 2010). Molecular phylogenetic analyses also recover *Shenzhousia* as a distinct lineage from both *Sinomytilus* and *Limnoperna* (Figs 2, 3).

##### Distribution.

This genus is currently known from the Yangtze River drainage and the Yellow River basin in China, including records from Kweifoo (present-day Fengjie, Chongqing), the Yuanjiang River, Changde, Hunan Province, and the Ba River, Xi’an, Shaanxi Province.

#### Shenzhousia swinhoei

Taxon classificationAnimaliaMeganisopteraMeganeuridae

(H. Adams, 1870) comb. nov.

CDE5557A-2CD7-5C46-B29D-82C7473F2E5D

Dreissena swinhoei H. Adams, 1870: 379, pl. 27, fig. 13. Type locality: on rocks in the Yangtze River at Kweifoo [= Kuifu 夔府, present-day Fengjie, Chongqing], China.
Sinomytilus swinhoei (H. Adams, 1870): Graf 2013: 152.

##### Neotype designation.

The original type material of *Dreissena swinhoei* H. Adams, 1870 could not be located despite searches in relevant historical collections and is presumed lost. To stabilise the taxonomic usage of this name, especially in the present generic reassignment, we herein designate specimen NCUMB.CDSS2601 as the neotype (Fig. 8B–E).

##### Material examined.

***Neotype*** (here designated): • NCUMB.CDSS2601, collected from Yuanjiang River, Changde, Hunan Province, 28.963545°N, 111.784480°E, deposited in Museum of Biology, Nanchang University (NCUMB), China; • NCUMB.XASS2609, Collected from Ba River, Xi’an, Shaanxi Province, China, 34°25'19"N, 109°00'43"E, deposited in Museum of Biology, Nanchang University (NCUMB), China.

##### Diagnosis.

Shell medium-sized, strongly inequilateral, axe-shaped; anterior end sharply tapering, posterior margin broadly rounded; umbo terminal to subterminal, prominent; a distinct ridge extending from umbo to posteroventral extremity; hinge edentulous, septum weak and inconspicuous; anterior adductor muscle absent.

##### Description.

Shell medium-sized (9.79–24.65 mm), relatively solid, distinctly inequilateral, strongly heteromyarian, outline elongate wedge-shaped to narrowly triangular, moderately inflated, with a characteristic axe-shaped profile. Anterior extremity sharply tapering and acute; posterior margin broadly rounded. Umbo terminal to subterminal, prominent, strongly elevated, and acute. Dorsal margin nearly straight to weakly arcuate, gently sloping posteriorly; ventral margin distinctly concave, especially in the anterior half. A single well-developed keel originates from the umbonal region and extends continuously to the posteroventral extremity, defining the characteristic axe-shaped shell outline (Fig. 8A–E).

Shell surface smooth and glossy, externally yellowish olive to light brown, bearing fine, closely spaced concentric growth lines and occasional dark purplish growth bands; radial sculpture absent. Periostracum thin. Under SEM, the shell surface additionally shows slight longitudinal striations (Fig. 7G–I).

Interior smooth and glossy, pale silvery to bluish gray, with a weak nacreous luster; anterior adductor muscle strongly reduced, no anterior adductor muscle scar visible; posterior adductor muscle scar subovate and shallow. Septum weak and inconspicuous.

Hinge edentulous. Ligament narrow and externally inconspicuous, situated along the anterodorsal margin immediately posterior to the umbo.

##### Habitat and distribution.

Originally recorded from rocks in the Yangtze River at Kweifoo, China (Adams 1870). Newly collected specimens were obtained from the Ba River, Xi’an, Shaanxi Province, and the Yuanjiang River, Changde, Hunan Province, indicating that the species is currently known from both the Yellow River basin and the Yangtze River drainage in China. It inhabits shallow freshwater environments at depths of ~ 2–5 m, where individuals are attached by byssal threads to submerged stones on the river bottom.

##### Remarks.

The newly collected material is here considered conspecific with the original concept of *Dreissena swinhoei* H. Adams, 1870. This interpretation is supported by the fact that both the type locality and part of the newly collected material are distributed within the Yangtze River drainage, and the newly collected specimens show a high degree of morphological concordance with the original description and illustration, particularly in relation to its shell outline, the prominent umbonal region, and the distinct continuous ridge extending from the anterior region to the posteroventral extremity.

Furthermore, the stable occurrence of this species in both the Yangtze River drainage and the Yellow River basin suggests that it is not a narrowly endemic taxon, but rather may possess a broader and geographically continuous distribution across major freshwater systems of China. This broader distribution pattern further supports the interpretation that the newly collected material represents the same species as that described in the original literature.

The designation of a neotype in the present study serves to stabilise the taxonomic usage of the name *Dreissena swinhoei* and provides a fixed modern reference for its identity, thereby facilitating its reassignment to *Shenzhousia* gen. nov. and future comparative studies.

## Discussion

Notable differences in shell morphology and anatomical characters distinguish Chinese *Sinomytilussensu lato* from the Southeast Asian *Sinomytilus harmandi*, the type species of the genus. Previous studies have suggested that *Sinomytilus* may not be monophyletic, but these analyses were based on limited sampling or single-gene data, leaving the generic boundaries of this group unresolved (Sokolova et al. 2021; Tan and Tan 2024; Wang et al. 2024). In this study, we sampled populations of *Sinomytilussensu lato* throughout their range in China, including Lake Fuxian, the Luliang palaeolake basin, and major river systems. Our findings demonstrate that *Sinomytilus* as currently defined is polyphyletic. *Sinomytilus swinhoei* from the Yangtze River system and Yellow River basin clusters with *Limnoperna* species rather than with *Sinomytilus harmandi*. Therefore, we propose the establishment of a new genus: *Shenzhousia* gen. nov., with *Sinomytilus swinhoei* as the type species. The subfamily Limnoperninae, within which these freshwater lineages are placed, forms a well-supported clade containing *Sinomytilus*, *Shenzhousia*, *Shanomytilus* and *Limnoperna*, consistent with the revised classification of Mytiloidea (Tan and Tan 2024; Bolotov et al. 2026). The variation in anterior adductor development among the sampled freshwater modiolids does not appear to follow a simple linear evolutionary trend. Instead, the presence of a relatively well-developed anterior adductor in *Shanomytilus* and *Limnoperna*, contrasted with its strong reduction in *Sinomytilus* and *Shenzhousia*, suggests that reduction of this muscle likely occurred independently in the lineages leading to *Sinomytilus* and *Shenzhousia*, possibly in association with the evolution of their highly specialised axe-shaped shell morphology and anterior compression. This pattern of convergent reduction is consistent with the phylogenetic results presented above, which demonstrate that *Sinomytilussensu lato* is polyphyletic. The independent loss or reduction of the anterior adductor in multiple lineages may reflect similar selective pressures associated with burrowing or byssal attachment in different freshwater habitats.

Divergence time estimates for freshwater modiolids in previous studies (e.g. Sokolova et al. 2021) are generally younger than those recovered here, which is likely attributable to differences in analytical strategies rather than true biological discrepancies. In the present study, divergence times were inferred using a concatenated multi-locus dataset (COI, 16S, 18S, 28S and H3) under a relaxed molecular clock framework in BEAST, with multiple fossil calibration points applied across different phylogenetic depths. Compared with approaches based on more limited datasets, the incorporation of both mitochondrial and nuclear markers can better accommodate rate heterogeneity among lineages and reduce biases associated with single-locus analyses. In addition, the use of multiple, well-distributed calibration points provides a more stable temporal framework for estimating node ages. Taken together, these methodological differences may explain the comparatively older divergence times recovered in this study, which are therefore considered to provide a more robust estimate of the evolutionary history of freshwater modiolid lineages. Molecular dating indicates that the sampled freshwater modiolid clade, comprising *Shanomytilus*, *Shenzhousia*, *Sinomytilus*, and *Limnoperna*, diverged from its nearest sampled marine relative at a mean age of ca 173.39 Ma. However, this estimate is associated with a broad 95% highest posterior density (HPD) interval ranging from 99.35 to 264.1 Ma, reflecting considerable temporal uncertainty that spans from the late Paleozoic to the mid-Cretaceous. While the mean estimate aligns closely with the middle Jurassic, a period characterised by pronounced environmental instability around the Aalenian–Bajocian (Hallam 2001; Haq 2017), the wide age range necessitates a cautious interpretation of the exact timing of this transition. Given that these taxa and *Leiosolenus* are currently resolved as sister groups (Figs 2, 3, 5), this node provides a valuable, albeit broad, temporal framework for the earliest sampled marine–freshwater split within this lineage. Such a timeframe encompasses intervals of significant sea-level oscillations and shoreline reorganisation, which may have fragmented shallow coastal habitats and generated isolated marginal basins. These environmental shifts likely provided the ecological stepping-stones necessary for marine ancestors of Modiolidae to progressively invade brackish and subsequently freshwater environments. Taken together, while the precise timing remains to be further refined, our results suggest that the emergence of the freshwater modiolid lineage represents a transition driven by long-term habitat fragmentation and repeated sea-level fluctuations during the Mesozoic. Subsequent tectonic uplift and large-scale freshwater system reorganisation in East and Southeast Asia during the later Mesozoic and Cenozoic may have further promoted lineage isolation and later diversification within the freshwater clade (Clark et al. 2004; Wang et al. 2020; Guo et al. 2021).

The ecological success and widespread distribution of *Limnoperna fortunei* provide a useful modern example for understanding the evolutionary capacity of freshwater modiolids. Native to East Asia but now widely introduced across multiple continents, this species has achieved rapid expansion largely through anthropogenic dispersal pathways such as ballast water and artificial waterways (Morton and Dinesen 2010; Morton 2015; Xiao et al. 2025). Its invasion success is underpinned by a combination of biological traits, including efficient byssal attachment to hard substrates, high reproductive output consistent with an r-selected life-history strategy, and strong physiological tolerance to varying freshwater conditions. As a result, *L. fortunei* is capable of forming dense aggregations that significantly alter benthic habitats, nutrient cycling, and trophic structure, while also causing severe biofouling in hydraulic systems (Morton 2015; Morton et al. 2020; Xiao et al. 2025). In light of our phylogenetic results, which indicate an early origin and long-term persistence of freshwater lineages within Modiolidae, the ecological success of *Limnoperna fortunei* should not be interpreted as evidence of widespread ecological equivalence among freshwater modiolids. Instead, it likely represents a lineage-specific expansion built upon an earlier evolutionary transition to freshwater environments. This suggests that while the shift from marine or brackish ancestors to freshwater habitats occurred early in the evolutionary history of the group, only certain lineages subsequently acquired the ecological traits necessary for large-scale dispersal and expansion.

Particularly noteworthy is the deep divergence of *Shanomytilus* at ca 89.37 Ma (Fig. 5; 95% HPD: 44.1–147.52 Ma). This placement within the Cretaceous (extending from the Late Jurassic to the eocene) suggests that this lineage represents an ancient and relict freshwater branch within Modiolidae. At present, *Shanomytilus* is known not only from Yunnan (Lake Fuxian and the Luliang palaeolake basin) but also from the Irrawaddy, Chao Phraya, and Mekong river basins in Myanmar and Thailand (Bolotov et al. 2026), suggesting a broader distribution across Indochina. The occurrence of the extinct subfossil species *S. frendae* in the Luliang palaeolake basin suggests that plateau lakes in southwestern China may have served as long-term refugia for ancient freshwater modiolid lineages. The conservation significance of these Yunnan plateau lake systems should also be emphasised. The Luliang palaeolake basin provides a striking example of how lacustrine habitat loss can erase highly endemic freshwater faunas. Previous studies have shown that the former Luliang lake group once supported a diverse molluscan assemblage, including multiple sympatric species of *Tchangmargarya*, other small endemic gastropods, as well as freshwater mussels, which has therefore been interpreted as a vanished Quaternary lacustrine biodiversity hotspot (Zhang and Wang 2023; He et al. 2025; Xiang et al. 2025, 2026). In contrast to the vanished Luliang lake system, Lake Fuxian still harbours living endemic freshwater faunas, including highly localized gastropod lineages and the extant species of *Shanomytilus* (Wang et al. 2024; Chen et al. 2025b; Zheng et al. 2026b). However, recent studies have indicated that the biodiversity of Lake Fuxian is threatened by environmental degradation, pollution, species invasion, and the restricted distributions of many endemic taxa (Cui et al. 2008; Zhang et al. 2024; Chen et al. 2025b; Zheng et al. 2026b). The apparent decline of native freshwater snail populations in Lake Fuxian, together with similar biodiversity losses observed in other Yunnan plateau lakes, highlights the vulnerability of ancient-lake ecosystems to anthropogenic disturbance. Therefore, the evolutionary uniqueness and restricted distributions of these freshwater modiolids underscore the urgent need for systematic biodiversity surveys, long-term monitoring, habitat protection, and pollution control in Yunnan plateau lakes.

## Supplementary Material

XML Treatment for Shanomytilus

XML Treatment for Shanomytilus frendae

XML Treatment for Shenzhousia

XML Treatment for Shenzhousia swinhoei

## Additional information

### Artificial Intelligence (AI) use

The authors accept full responsibility for the content of the manuscript, including the disclosure of any use of AI.

No AI tools were used in the preparation of this manuscript.

### Author ORCIDs

H. Zheng https://orcid.org/0009-0004-5222-3975

X.-X. Wang https://orcid.org/0009-0005-8009-4346

Y.-M. He https://orcid.org/0009-0002-9849-7977

H.-Q. Xiang https://orcid.org/0009-0005-6737-3092

X.-P. Wu https://orcid.org/0000-0002-8037-5640

### Data availability

The data underlying this article are available in GenBank or uploaded as Supporting Information available at ZooKeys online.

## Supplementary materials

Supplementary material 1 List of COI and 16S sequences used in this study. Sequences obtained for this study are marked by an asterisk Data type xlsx https://binary.pensoft.net/file/1814094This dataset is made available under the Open Database License (http://opendatacommons.org/licenses/odbl/1.0/). The Open Database License (ODbL) is a license agreement intended to allow users to freely share, modify, and use this Dataset while maintaining this same freedom for others, provided that the original source and author(s) are credited. Hui Zheng, Xing-Xiao Wang, Ji-Wang Xie, Chun-Hua Zhou, Yue-Ming He, Hong-Quan Xiang, Shan Ouyang, Fang-Fa Zeng, Xiao-Ping Wu

Supplementary material 2 List of COI, 16S, 18S, 28S, and H3 sequences used in this study. Sequences obtained for this study are marked by an asterisk Data type xlsx https://binary.pensoft.net/file/1814095This dataset is made available under the Open Database License (http://opendatacommons.org/licenses/odbl/1.0/). The Open Database License (ODbL) is a license agreement intended to allow users to freely share, modify, and use this Dataset while maintaining this same freedom for others, provided that the original source and author(s) are credited. Hui Zheng, Xing-Xiao Wang, Ji-Wang Xie, Chun-Hua Zhou, Yue-Ming He, Hong-Quan Xiang, Shan Ouyang, Fang-Fa Zeng, Xiao-Ping Wu

Supplementary material 3 List of sequences used in mitochondrial phylogenomic analyses Data type xlsx https://binary.pensoft.net/file/1814096This dataset is made available under the Open Database License (http://opendatacommons.org/licenses/odbl/1.0/). The Open Database License (ODbL) is a license agreement intended to allow users to freely share, modify, and use this Dataset while maintaining this same freedom for others, provided that the original source and author(s) are credited. Hui Zheng, Xing-Xiao Wang, Ji-Wang Xie, Chun-Hua Zhou, Yue-Ming He, Hong-Quan Xiang, Shan Ouyang, Fang-Fa Zeng, Xiao-Ping Wu

Supplementary material 4 Partitioning strategies from PartitionFinder2 according to AICc Data type xlsx https://binary.pensoft.net/file/1814097This dataset is made available under the Open Database License (http://opendatacommons.org/licenses/odbl/1.0/). The Open Database License (ODbL) is a license agreement intended to allow users to freely share, modify, and use this Dataset while maintaining this same freedom for others, provided that the original source and author(s) are credited. Hui Zheng, Xing-Xiao Wang, Ji-Wang Xie, Chun-Hua Zhou, Yue-Ming He, Hong-Quan Xiang, Shan Ouyang, Fang-Fa Zeng, Xiao-Ping Wu
